# Gene expression trend changes in breast cancer populations over two decades: insights from The Cancer Genome Atlas database

**DOI:** 10.1186/s41065-022-00230-3

**Published:** 2022-03-22

**Authors:** Jinbo Wu, Hongjun Liu, Taobo Hu, Shu Wang

**Affiliations:** Department of Breast Surgery, Peking University People’s Hospital, Beijing, China

**Keywords:** Breast cancer, Gene expression, TCGA

## Abstract

**Background:**

Breast cancer has remained the most common malignancy in women over the past two decades. As lifestyle and living environments have changed, alterations to the disease spectrum have inevitably occurred in this time. As molecular profiling has become a routine diagnostic and objective indicator of breast cancer etiology, we analyzed changes in gene expression in breast cancer populations over two decades using The Cancer Genome Atlas database.

**Methods:**

We performed Heatmap and Venn diagram analyses to identify constantly up- and down-regulated genes in breast cancer patients of this cohort. We used Kyoto Encyclopedia of Genes and Genomes (KEGG) enrichment analyses to visualize associated functional pathways.

**Results:**

We determined that three oncogenes, *PD-L2*, *ETV5*, and *MTOR* and 113 long intergenic non-coding RNAs (lincRNAs) were constantly up-regulated, whereas two oncogenes, *BCR* and *GTF2I*, one tumor suppression gene *MEN1*, and 30 lincRNAs were constantly down-regulated. Up-regulated genes were enriched in “focal adhesion” and “PI3K-Akt signaling” pathways, etc., and down-regulated genes were significantly enriched in “metabolic pathways” and “viral myocarditis”. Eight up-regulated genes exhibited doubled or higher expression and the expression of three down-regulated genes was halved or lowered and correlated with long-term survival.

**Conclusions:**

In this study, we found that gene expression and molecular pathway enrichments are constantly changing with time, importantly, some altered genes were associated with prognostics and are potential therapeutic targets, suggesting that the current molecular subtyping system must be updated to keep pace with this dynamic change.

## Introduction

Globally, breast cancer has the highest incidence among all cancers, surpassing lung cancer. In 2020, this disease had an estimated 2.3 million new cases, representing approximately 11.7% of all new cancers [1]. Breast cancer incidence rates are increasing annually [2] and may be due to several key carcinogenic and breast cancer progression factors including, hormonal risk factors (early menarche, late menopause, advanced age at first birth, fewer children being born, lower breastfeeding rates, hormone therapy for the menopause, oral contraceptive use, Vitamin D, and thyroid hormone deficiency), lifestyle risk factors (alcohol intake, excess body weight, physical inactivity, smoking, and antibiotic use), genetic factors (family history of disease and high-penetrance genes), environmental factors (elevated reactive oxygen species levels, higher airborne heavy metals, synthetic chemicals, and radiation), and increased screening [2–9].

Breast cancer detection and intervention at early stages is key in improving prognoses and reducing mortality rates. In the past two decades, researchers have used several conventional and novel breast cancer diagnostic approaches, including mammography, magnetic resonance imaging, ultrasound, biopsies, serum screening for (microRNAs) miRNAs, blood-based proteomics, biomarker analyses, and biosensor technologies [10–12]. Based on evidence-based medicine, the comprehensive treatment of breast cancer primarily involves surgery combined with chemotherapy, endocrine therapy, radiation therapy, and targeted therapies [11–13]. In recent years, thanks to advances in genetic sequencing techniques, management strategies for malignant tumors have entered a new era of molecular medicine and precise treatment [14–16]. Molecular classification, targeted therapy, and immunotherapy approaches aimed at specific genes have considerably ameliorated treatment responses, overall survival (OS), and disease-free survival rates in patients with the disease [17–20]. However large-scale prospective studies comprising thousands of individuals can take 5–10 years to reach definitive conclusions, the lagging-behind findings have some limitations and defects [21–23].

Guidelines on breast cancer screening and diagnosis strategies exert profound effects on breast cancer diagnostics and treatment. One particular, pressing issue relates to whether breast cancer patients diagnosed today are identical or similar to those diagnosed decades ago in terms of clinicopathological characteristics and molecular biological features. This concept is not unusual and is seen in other disciplines such as infectious diseases and climate adaptation. As time progresses, the spectrum of diseases threatening human health is constantly, and indeed, inevitably changing. Globally, at the beginning of the twentieth century, infectious and parasitic diseases were the leading cause of death, however, this status has changed to chronic and degenerative diseases [24, 25]. Climate change is also associated with changes in infectious disease epidemiology; it is predicted that populations at risk for diarrheal disease, malnutrition, and malaria will increase if global warming continues [26–28]. Similar studies have been performed for breast cancer; the incidence of estrogen receptor (ER) positive breast cancer has increased slightly for nearly 20 years [29–31]. Database analyses have shown that the risk from different types of breast cancer has varied in women of different ages and ethnicities, and has changed over time and not remained static. Yet, gene profiles reflecting breast cancer changes over time have not been reported, therefore, are contemporary gene expression profiles for breast cancer consistent with profiles from 10 or 20 years ago? This question has serious implications for drug development, screening, and therapeutic strategies, therefore, scientists and clinicians rethink and redefine the value of long-standing evidence-based guidelines in guiding clinical practice for emerging diseases. To address this knowledge gap, we used The Cancer Genome Atlas (TCGA) database to generate a preliminary analysis.

## Results

### Up- and down-regulated genes in breast cancer patients

**Fig. 1:**
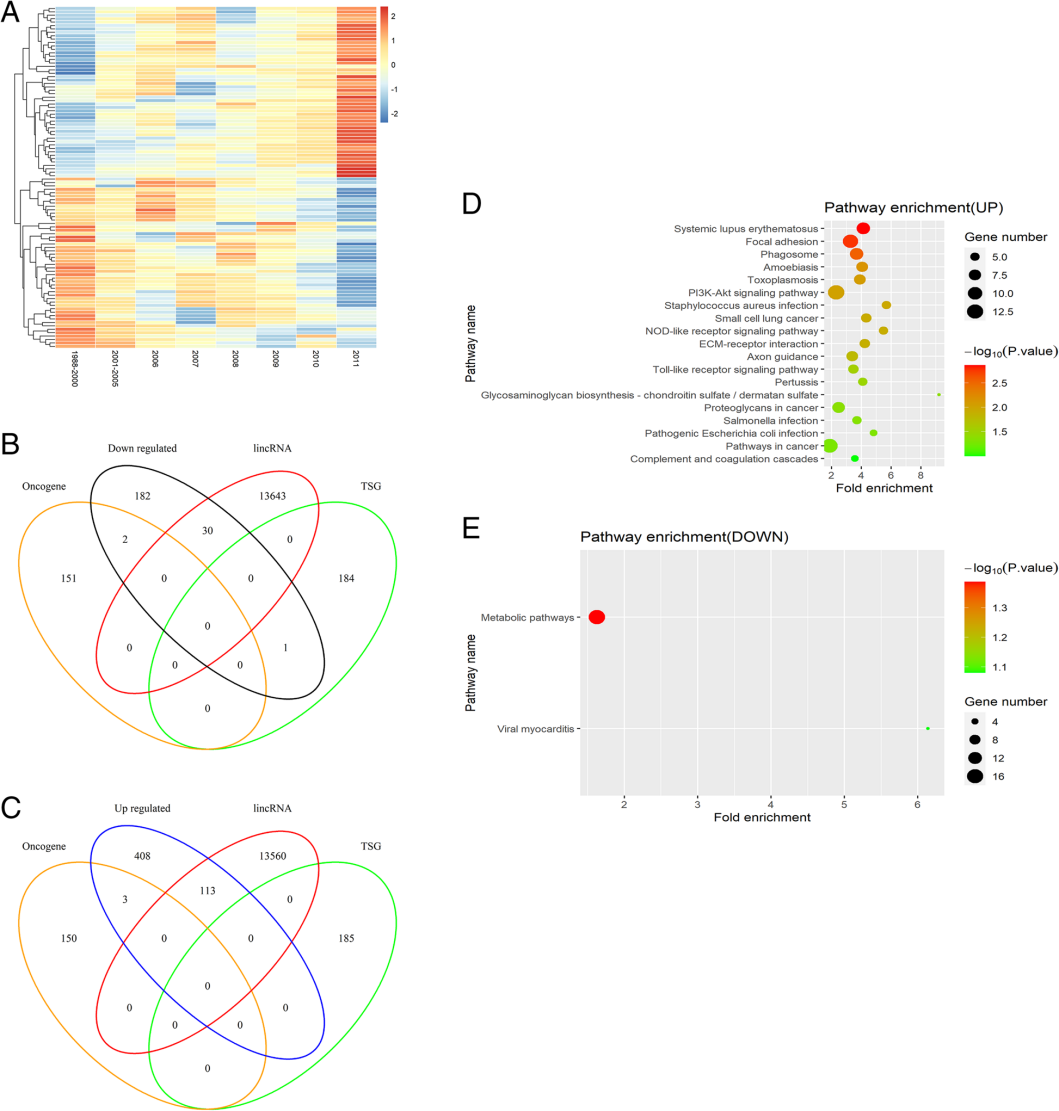
Identification of altered genes and associated KEGG analyses from eight groups. **A** Heatmap showing the top 50 up-regulated and top 50 down-regulated genes from the eight groups. Red = up-regulated; Blue = down-regulated. The expression intensity value is derived from gene expression levels using R software analysis. **B** Venn diagram showing shared genes between down-regulated genes, recognized oncogenes, lincRNAs, and TSGs. **C** Venn diagram showing shared genes between up-regulated genes, recognized oncogenes, lincRNAs, and TSGs. **D** KEGG pathway results of the up-regulated genes. The size of each circle represents the gene number in the corresponding pathway, which is proportional to the circles in the caption. **E** KEGG pathway results for down-regulated genes; the x-axis represents fold enrichment, different colors represent −log10 (*P*-value), and circle sizes represent gene numbers in a specific pathway. Abbreviations: lincRNA, long intergenic non-coding RNA; TSG, tumor suppressor gene; KEGG, Kyoto Encyclopedia of Genes and Genomes

We identified 524 up-regulated and 215 down-regulated genes in 1102 patients. Patients diagnosed with breast cancer between 1988 and 2011 were classified into eight groups according to the year of diagnosis. A heatmap of the top 50 up-regulated and 50 down-regulated genes from eight groups was generated (Fig. 1A; red = up-regulated and blue = down-regulated genes). When we compared the 2011 group with the initial 1998–2000 group, the top five up-regulated genes with the largest log2 fold-change in expression were; *AC007728.3*, *AC097460.1*, *AC010542.4*, *USP50*, and *BX276092.9*, at 2.5, 2.3, 2.2, 2.1, and 2.0, respectively. The top five down-regulated genes with the largest log2 fold-change in expression were; *C1QTNF9*, *AC011479.1*, *MTND4LP30*, *KRTDAP*, and *AP000251.1*, at 1.8, 1.7, 1.3, 1.2, and 1.1, respectively. We observed two oncogenes *BCR* and *GTF2I*, one tumor suppression gene (TSG), *MEN1*, and 30 long intergenic non-coding RNAs (lincRNAs) in down-regulated genes (Fig. 1B). Notably, the log2 fold-change in BCR expression was 0.2, with a significant Kaplan-Meier *P*-value of 0.02. We also identified three oncogenes, *PD-L2*, *ETV5*, and *MTOR*, and 113 lincRNAs in up-regulated genes (Fig. 1C). Likewise, the log2 fold-change in *PD-L2* expression was 0.9, but with a borderline significant Kaplan-Meier *P*-value of 0.06. Additionally, we analyzed four genotyping groups. In 232 patients in the Luminal A group, 665 up-regulated and 553 down-regulated genes were identified. *CST1* displayed the largest log2 fold-change in up-regulated expression (3.1), and *MPPED1* had the second-largest log2 fold-change decrease at 2.9. The up-regulated genes in Luminal B (125 patients), basal-like (101 patients), and HER2-enriched (58 patients) groups were 637, 668, and 500, respectively, and the highest log2 fold-change genes were *IGHV3-20* (8.8), *NDUFA5P11* (5.8), and *HNRNPA1P26* (6.4), respectively. Also, down-regulated genes in Luminal B, basal-like, and HER2-enriched groups were 547, 800, and 615, respectively; the highest log2 fold-change genes were *PLA2G3* (4.2), *TRAV18* (5.0), and *AL390294.1* (9.7), respectively (Table 1).

**Table 1:** Identification of up-and down-regulated genes in breast cancer patients of four intrinsic subtypes

Subtype	Total patients	up-regulated genes	down-regulated genes
Luminal A	232	665	553
Luminal B	125	637	547
basal-like	101	668	800
HER2-enriched	58	500	615

### Significantly enriched Kyoto encyclopedia of genes and genomes (KEGG) pathways

**Fig. 2:**
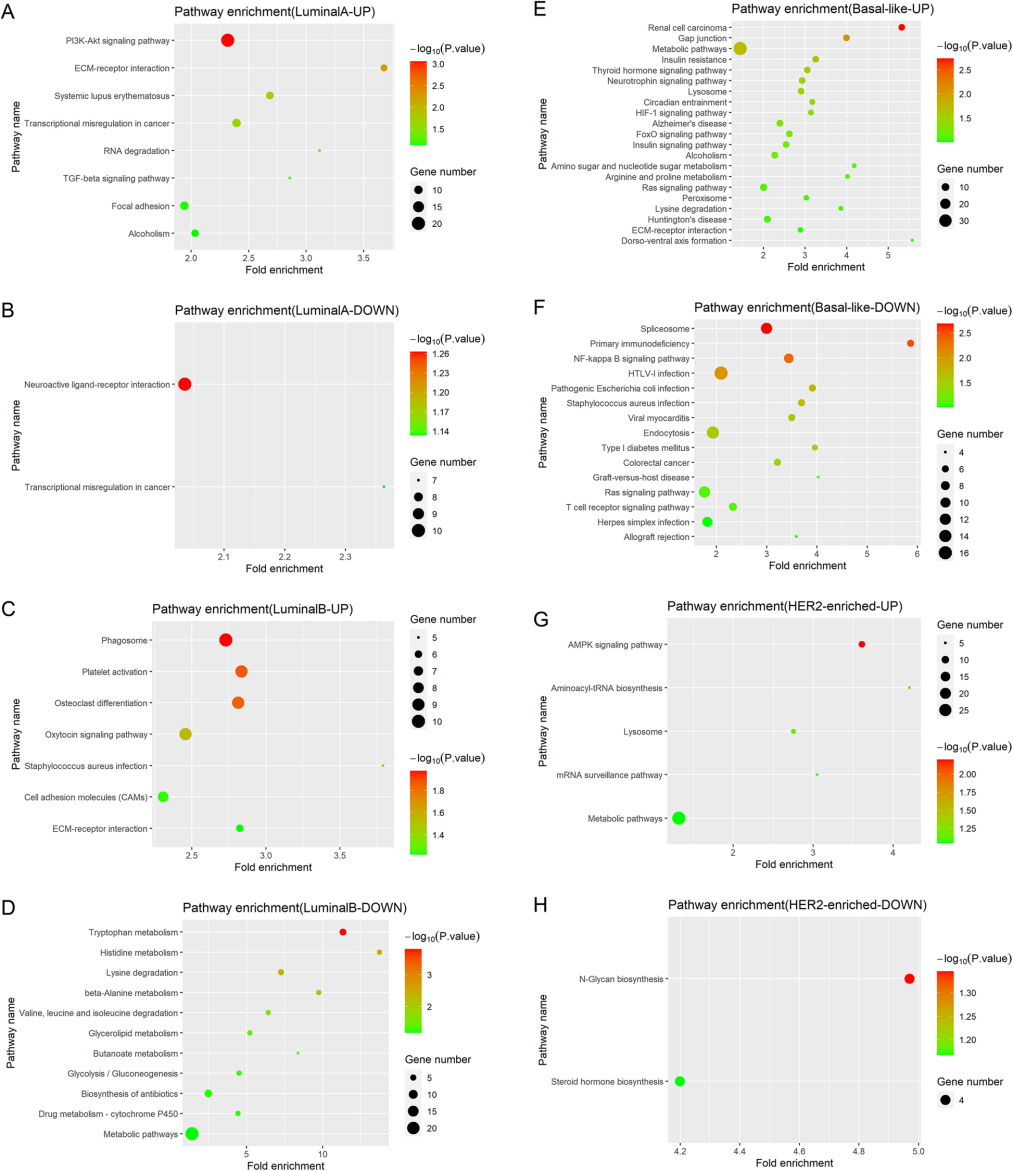
KEGG pathway analyses of four intrinsic subtypes containing up-regulated and down-regulated genes. **A** Up-regulated genes in the Luminal A group; **B** Down-regulated genes in the Luminal A group; **C** Up-regulated genes in the Luminal B group; **D** Down-regulated genes in the Luminal B group; **E** Up-regulated genes in the basal-like group; **F** Down-regulated genes in the basal-like group; **G** Up-regulated genes in the HER2-enriched group; **H** Down-regulated genes in the HER2-enriched group; the x-axis indicates fold enrichment, different colors represent −log10 (*P*-value), and circle sizes represent gene numbers in a specific pathway. Abbreviations: KEGG, Kyoto Encyclopedia of Genes and Genomes

Up-regulated and down-regulated genes were uploaded separately. Up-regulated genes were enriched in 19 pathways, including “focal adhesion”, “PI3K-Akt signaling”, “NOD-like receptor signaling”, “ECM-receptor interaction”, “Toll-like receptor signaling”, etc. Down-regulated genes were significantly enriched in two pathways; “metabolic” and “viral myocarditis” (Fig. 1D, E). The overlapping gene sets in pathways were *ITGB1*, *ITGA4*, *ACTN1*, *ROCK1*, *MTOR*, *CD80*, etc. Interestingly, 10 pathways were enriched in the Luminal A group; “PI3K-Akt signaling” had 20 up-regulated genes, most significantly (*p* < 0.001, Fig. 2A, B). “ECM-receptor interaction” was immediately followed (*p* = 0.006). The Luminal B group had seven enriched pathways containing up-regulated genes and 11 pathways containing down-regulated genes (Fig. 2C, D). These encompassed “phagosome”, “platelet activation”, “osteoclast differentiation”, “oxytocin signaling”, “tryptophan metabolism”, “histidine metabolism”, “lysine degradation”, “β-alanine metabolism”, etc. As shown (Fig. 2E, F), 21 enriched pathways were identified in the basal-like group containing up-regulated genes and 15 pathways containing down-regulated genes. For instance, “Ras signaling”, “metabolic”, “insulin signaling”, “thyroid hormone signaling”, “neurotrophy signaling”, “HIF-1 signaling”, “primary immunodeficiency”, and “type I diabetes mellitus”. Furthermore, the HER2-enriched group had five enriched pathways containing up-regulated genes and two pathways containing down-regulated genes (Fig. 2G, H). The “AMPK signaling” and “N-glycan biosynthesis” pathways were the most interesting, with eight up-regulated and four down-regulated genes, respectively.

### Survival analysis and expression trends of hub genes

**Fig. 3:**
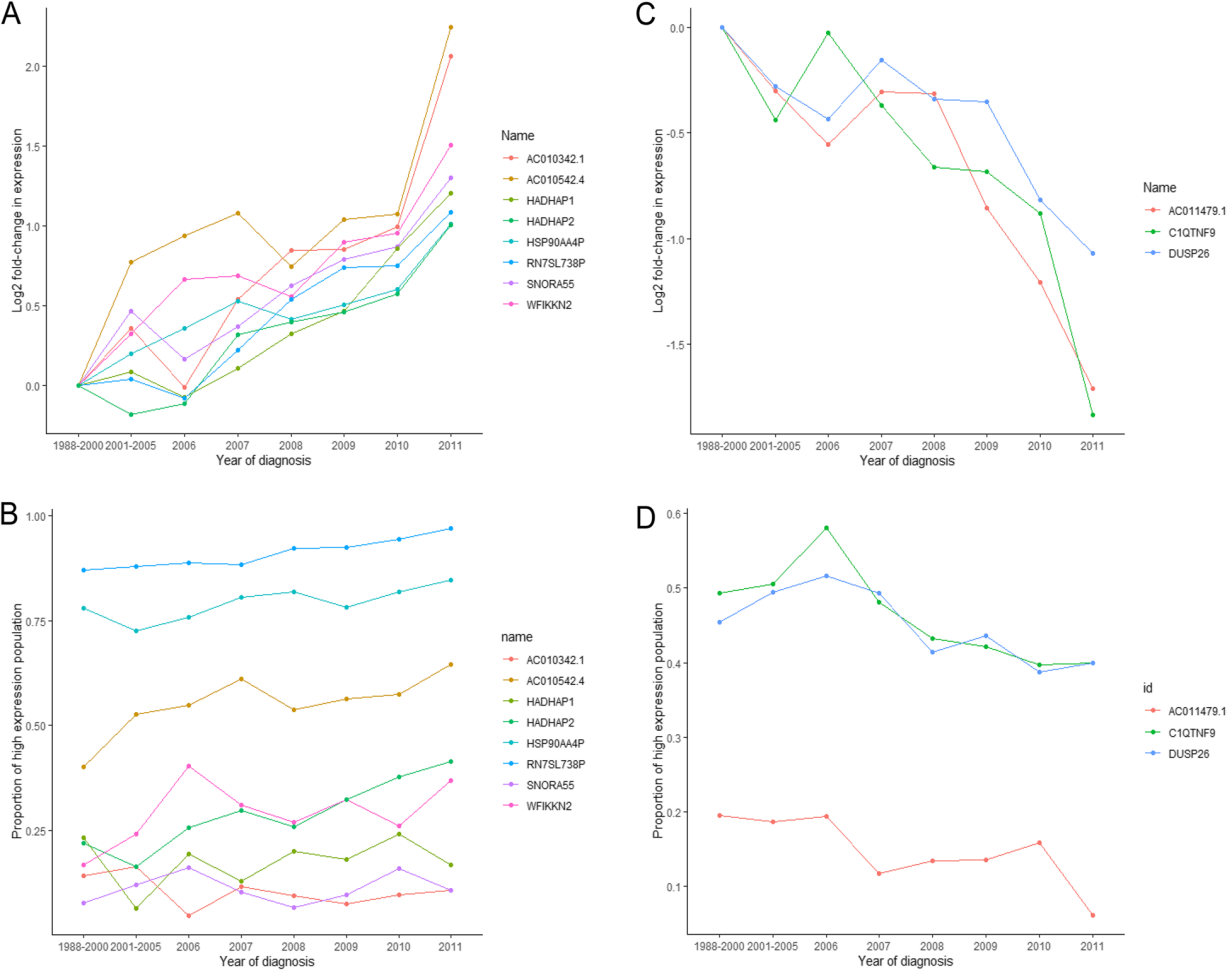
Line charts of log2 fold-changes in the expression of hub up-regulated (**A**) and down-regulated genes (**C**) in the eight groups in the TCGA-BRCA database. Line charts of the proportion of high expression population of hub up-regulated (**B**) and down-regulated genes (**D**). Abbreviations: TCGA = The Cancer Genome Atlas; BRCA = breast cancer

**Fig. 4:**
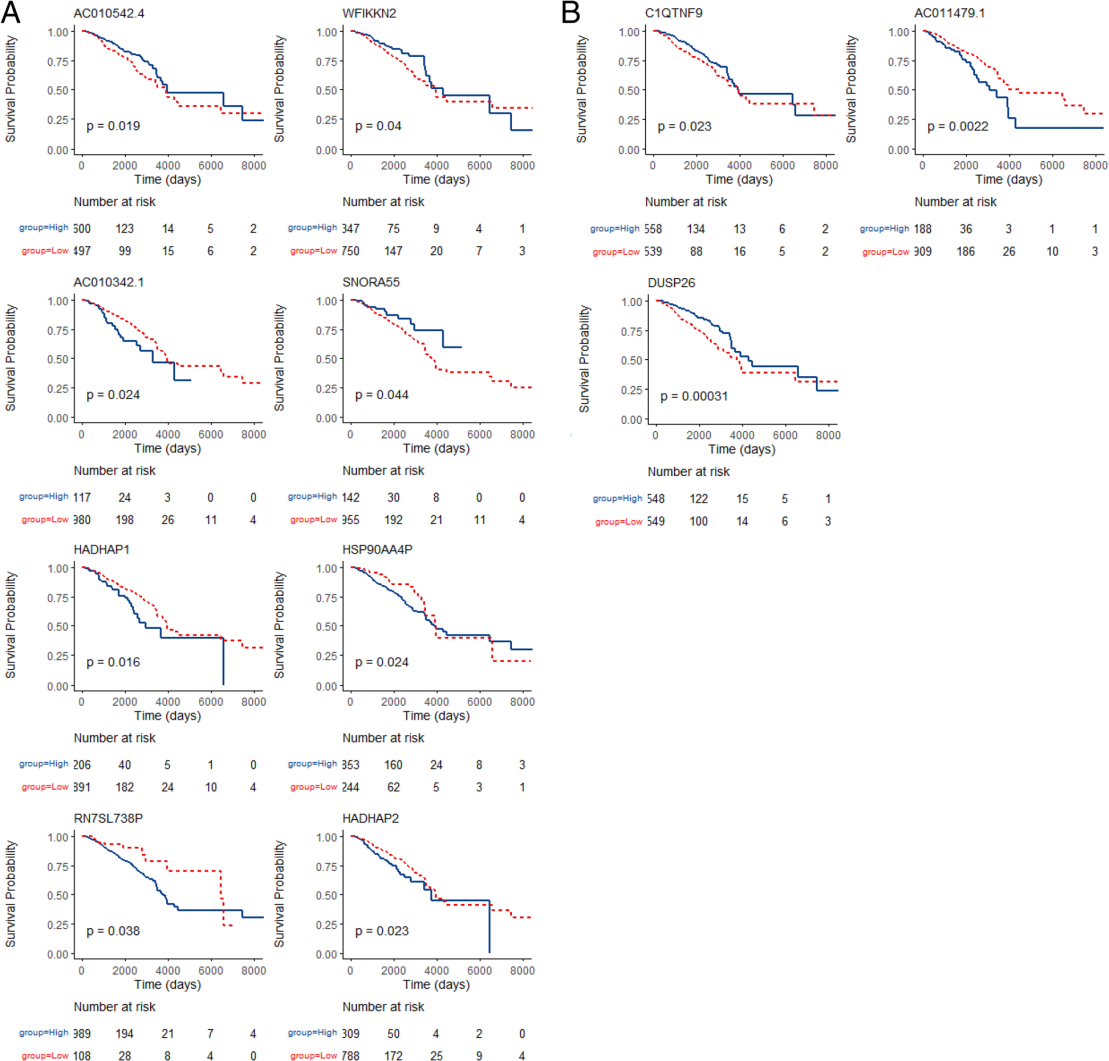
Kaplan-Meier plots of high and low groups stratified by expression values of hub up-regulated (**A**) and down-regulated genes (**B**); blue lines = high expression groups and red lines = low expression groups

As shown in Fig. 3, eight up-regulated and three down-regulated genes were recognized as hub genes, which satisfied the following conditions; they did not belong to lincRNAs, they had a Kaplan-Meier *P*-value < 0.05, they had a log2 fold-change in expression > 1 when comparing the 2011 group with the 1998–2000) group, one drop allowed but the change in log2 expression less than one-third of the total change (2011 vs. 1988-2000). As shown in Fig. 4, elevated *WFIKKN2*, *SNORA55*, *C1QTNF9*, and *DUSP26* expression displayed significantly improved OS rates and longer median survival times. Also, lower *HSP90AA4P*, *HADHAP1*, *HADHAP2*, and *RN7SL738P* expression significantly extended patients’ lifespan. Moreover, we hypothesized that *USP50*, *IGLC6*, and *NACA2* genes, which had log2 fold-expression increases of 2.1, 1.8, and 1.0, respectively, showed potential to become novel clinical outcome predictors and therapeutic targets. Also, the number of people with higher expression had been increasing by about 10% in the last 20 years. In the same way, *C1QTNF9* and *DUSP26* had log2 fold-decreases in the expression of 1.8 and 1.1 (Fig. 3C), and the number of people with higher expression had been decreasing by about 10% in the last 20 years (Fig. 3D), respectively.

## Discussion

Over the past 20 years, we observed that > 700 genes had changed and were enriched in “PI3K-Akt signaling”, “ECM-receptor interaction” and “Toll-like receptor signaling”, etc. In different molecular disease groups, enriched pathways containing up-and down-regulated genes were different. For example, “PI3K-Akt signaling” in Luminal A, “phagosome” in Luminal B, “Ras signaling” in basal-like, and “AMPK signaling” in the HER2-enriched group. In addition, 11 genes were > 2-fold altered, were associated with a degree of survival prognosis (*p* < 0.05), and potentially functioned as therapeutic targets.

Precision medicine has become an essential part of cancer treatment. Targeted molecular therapies and immunotherapies are rapidly moving toward an era of bespoke, precision medicine. Endocrine therapy for ER-positive patients in the 1980s [32, 33] and trastuzumab treatment for HER2-positive patients at the start of this century [34, 35] inaugurated targeted therapies for solid tumors. Surgery, radiotherapy, endocrine therapy, chemotherapy, and/or targeted therapies based on molecular subtyping have also paved the way for “precision medicine” for breast cancer. Additionally, risk prediction models, including the 21-gene assay (Oncotype DX Recurrence Score) and 70-gene assay (commercially known as Mammaprint) have become familiar in clinical settings to provide guidelines for systemic chemotherapy efficacy, and also endocrine therapy which may de-escalate chemotherapy [36–38]. Similarly, the inception of gene profiling and next-generation sequencing has meant precision medicine is now closer to clinical practice. Specifically, for ER-positive patients with endocrine therapy resistance, omics-data studies have uncovered mechanisms underpinning “CDK4/6 signaling” and “PI3K-Akt signaling” implicated in tumorigenesis and drug resistance. Similarly, prospective clinical trials also confirmed that the CDK4/6 inhibitor, palbociclib, the mTOR inhibitor, everolimus, and the PI3K inhibitor, buparlisib may improve progression-free survival (PFS) in patients with advanced breast cancer [39–42]. Also, “MAPK signaling” and “PI3K-Akt signaling” activation are closely associated with tumor cell proliferation in HER2-positive patients. Several clinical trials have explored the efficacy of PI3K inhibitors and tyrosine kinase inhibitors in overcoming resistance to anti-HER2 therapy [43, 44]. In triple-negative breast cancer, immune checkpoint inhibitors may have clinical applications due to “Ras signaling” activation and the elevated expression of immune-related genes such as PD-1, PD-L1, and CTLA-4 [45, 46]. Also, the poly ADP-ribose polymerase (PARP) inhibitors, Olaparib, and talazaparib can prolong PFS and improve patient quality of life in metastatic breast cancer caused by germline BRCA mutations [47–49].

We observed that *USP50*, *GPR174*, *HADHAP2*, *NACA2*, and *IGFBPL1* showed large expression changes, significant Kaplan-Meier *P* values, and increasing proportions in the population. We propose these molecules may serve as potential breast cancer therapeutic targets in the future. Aressy et al. proposed that *USP50* repressed activation of DNA damage checkpoints via an HSP90-dependent mechanism, leading to tumors [50]. Smith et al. reported that *IGFBP-rP1* and *IGFBPL1* expression was regulated by aberrant hypermethylation in breast cancer pathogenesis and that these genes may be beneficial in clinical practice [51]. In the Luminal A group, *CST1* exhibited the largest log2 fold expression increase (3.1); a previous study suggested *CST1* may function as a significant prognostic indicator and breast cancer therapeutic target [52]. Also, *ERBB4* expression exhibited a log2 fold-decrease of 3.7 in the HER2-enriched group, therefore *ERBB4* overexpression could have biological and prognostic significance for breast cancer [53].

Interestingly, 113 lincRNAs (21.6%) were up-regulated and 30 (14.0%) down-regulated in our study. Previous research indicated that lincRNAs regulate gene expression at epigenetic and transcription levels, and when the expression is altered, they promote cancer initiation and metastasis. Currently, several lincRNAs are significantly correlated with a cancer diagnosis, prognosis, and the therapeutic development of multitype cancers [54–56]. Our data indicated that several lincRNAs could function as potential prognostic biomarkers and have important clinical value, e.g., *RFPL1S*, *ADAMTS9-AS2*, *IBA57-AS1*, and *MYOSLID* are up-regulated lincRNAs [57–62] and *MORF4L2-AS1*, *LINC01278*, and *LINC00562* [63–67] are down-regulated. Importantly, all are related to the occurrence and development of several tumors by modulating “PI3K-Akt signaling”, “interferon type II signaling” and the expression of particular genes.

We also identified considerable changes in the “*Staphylococcus aureus*”, “Salmonella” and, “pathogenic *Escherichia coli*” infection pathways, which we suspect may be related to antibiotics overuse. Recent studies reported associations between antibiotic use and breast cancer risk via effects on inflammation, immune function, and estrogen and phytochemical metabolism [9]. Friedman et al. reported that in 2.1 million women followed up for 9 years, the use of any antibiotic was related to a slightly increased risk of developing breast cancer [Hazard ratio = 1.14; 95% confidence interval: 1.10–1.18] [68]. However, Basso et al. reported that ansamycin may be a beneficial HER2-positive breast cancer treatment by inhibiting the “Akt dependent pathway” and cyclin D expression [69]. We observed that the “focal adhesion pathway” changed considerably and was enriched by *ITGB1*, *ITGA4*, and nine other up-regulated genes. Strelnikov et al. claimed a strong association between abnormal *ITGA4* and *ITGB1* hypermethylation and HER2-positive tumors [70]. Previous studies indicated that microenvironment-related pathways, such as “focal adhesion”, “ECM-receptor interaction”, and “complement and coagulation cascades” identified in this study are closely related to tumor initiation, disease progression, and metastasis, which are important future research directions [71, 72]. In addition, we identified significant changes in metabolism-related pathways, especially in the Luminal B group, such as “glycosaminoglycan biosynthesis”, “proteoglycans in cancer”, “tryptophan metabolism”, and “β-alanine metabolism”. We hypothesize these pathways are associated with dietary intake and improvements in living standards [73]; encouraging results from animal studies and clinical trials revealed the clinical relevance of these pathways and the benefit of targeted drugs for cancer [74–77]. Interestingly, Budczies et al. reported that β-alanine accumulated in breast cancer tissues, especially in the ER-negative subtype, in agreement with our results [78].

Notably, we observed eight up-regulated genes in “AMPK signaling” in HER2-enriched patients, whose activity may retard the growth of several cancers. Jhaveri et al. showed that AMPK regulated HER2 activity in HER2-enriched breast cancer cells, therefore AMPK activation may elicit a therapeutic benefit for such cancers [79, 80]. The “alcoholism pathway”, enriched in Luminal A, suggested an elevated risk for breast cancer. Recent evidence suggested that every alcohol unit/day enhanced the possibility of breast cancer by 7–11%, and this process was mechanistically underpinned by increased estrogen levels, acetaldehyde, and oxidative stress [81, 82]. Research also showed that disulfiram, an anti-alcoholism drug used in the clinic, induced apoptosis in vitro breast cancer cells and showed potential therapeutic candidacy [83]. Beyond that, hormone dependence is a concerning issue; menopause hormone therapy and plasticizers used in daily life are closely associated with ER-pathway activation, potentially contributing to breast cancer [84–86]. The HABITS trial reported that estrogen and progestogen doses may be associated with breast cancer recurrence [87].

In this study, we determined that genes and molecular pathways are constantly changing, suggesting molecular typing technologies must keep pace with this dynamic situation. Therefore, new biomarkers or pathways must be explored based on traditional molecular types. Our study had many limitations; small sample size and short period. Also, our analyses may not have fully reflected influences from the environment, time, habits, and other factors. Similarly, our study was an exploratory, retrospective analysis and lacked external validation using other methods. Thus, to some extent, the effectiveness and representation of the TCGA database are limited. Nonetheless, ours is the first study to investigate tumor genomic changes from a historical perspective. Although limited, our work provides new research directions and instills debate on this key issue. The observation of dynamic tumor genomic changes has the potential to support and reinforce existing cancer prevention strategies, drug development research programs, and prognostic predictions.

## Materials and methods

### Data sources

The Cancer Genome Atlas-Breast Cancer (TCGA-BRCA) RNAseqV2 gene expression and clinical data were acquired from the TCGA data portal (https://cancergenome.nih.gov) [88]. The “SummarizedExperiment” Bioconductor package (http://www.bioconductor.org) was used to complete and normalize data files in R (version 4.0.0, R Foundation for Statistical Computing). RNA-seq data from TCGA-BRCA covering 57,035 protein-coding and non-coding genes were used for analysis. We included 1102 patients diagnosed with breast cancer between 1988 and 2011; they were classified into eight groups according to the year of diagnosis. Patients diagnosed between 1988 and 1989 and 2000 and 2005 were classified as two separate groups to balance patient numbers in each group. Gene names were annotated to “Ensemble-id” according to corresponding TCGA platform files.

### Gene identification

Expression changes of a particular gene in each group were defined as its average expression change in all breast cancers in that group. Genes whose expression levels were higher than those in the previous group, ≥ 6 times, were defined as up-regulated, and those whose expression levels were lower were defined as down-regulated. We produced heatmaps of the top 50 up-regulated and top 50 down-regulated genes identified in order of the log2 fold-change of the gene average expression of the last group (2011) to the initial group (1988-2000) using the “heatmap” package (version 2.7.7) in RStudio. Venn diagrams were also generated to identify up-regulated and down-regulated genes, known oncogenic genes, tumor suppressor genes (TSG) in the “OncoVar” database (https://oncovar.org), and known long intergenic non-coding RNA (lincRNA) in the “LNCipedia” database [89] using the “VennDiagram” package (version 1.6.20) in R.

### Kyoto encyclopedia of genes and genomes pathway enrichment analysis

KEGG is a practical database that contains molecular information used to predict pathways where particular genes are enriched [90]. KEGG enrichment analyses were performed using the Database for Annotation, Visualization, and Integrated Discovery (DAVID) (https://david.ncifcrf.gov). A *P* < 0.05 value was accepted as statistically significant. Breast cancers were classified into subtypes based on gene expression data. PAM50 breast cancer subtyping was widely used to classify breast cancer into four genotype groups: Luminal A, Luminal B, basal-like, and HER2-enriched [14]. We selected patients using the PAM50 subtypes from TCGA clinical information and the same analysis was performed for these four genotyping groups.

### Statistical analyses

Patients with breast cancer were assigned to high and low expression groups based on the auto best cutoff of up-regulated or down-regulated gene expression levels as calculated by “survminer” (version 0.4.8) and “survival” (version 3.1) packages in RStudio. OS was the time from the date of diagnosis to the date of death due to any cause, or the last follow-up date. The survival probability of high and low expression groups was calculated using the Kaplan-Meier method and compared using log-rank tests. We also used line charts to display log2 expression trends in altered genes, and the proportion of high expression population of changing genes during eight periods. Altered genes not belonging to lincRNAs, Kaplan-Meier *P* values < 0.05, log2 fold-change in expression > 1 when comparing the last group (2011) with the initial group (1998-2000), and one drop allowed but the change of log2 expression less than one-third of the total change (2011 vs. 1988-2000), are shown (Figs. 3 and 4).

## Conclusions

In conclusion, we analyzed changes in gene expression in breast cancer populations over two decades using the TCGA database. Our results proved that genes and molecular pathways are constantly changing, more importantly, some altered genes were associated with prognostics and are potential therapeutic targets. Our findings also suggest that the current molecular subtyping system of breast cancer should also be updated to keep pace with this dynamic situation.

## Data Availability

The original data used to support the findings of this study are available from TCGA Research Network (https://www.cancer.gov/tcga). The data sets and the R codes used in the current study are available from the corresponding author upon reasonable request.
